# Association between the TPMT*3C (rs1142345) Polymorphism and the Risk of Death in the Treatment of Acute Lymphoblastic Leukemia in Children from the Brazilian Amazon Region

**DOI:** 10.3390/genes11101132

**Published:** 2020-09-25

**Authors:** Darlen Cardoso de Carvalho, Luciana Pereira Colares Leitão, Fernando Augusto Rodrigues Mello Junior, Alayde Vieira Wanderley, Tatiane Piedade de Souza, Roberta Borges Andrade de Sá, Amanda Cohen-Paes, Marianne Rodrigues Fernandes, Sidney Santos, André Salim Khayat, Paulo Pimentel de Assumpção, Ney Pereira Carneiro dos Santos

**Affiliations:** 1 Oncology Research Nucleus, Universidade Federal do Pará, Belém 66063-023, Brazil; darlen.c.carvalho@gmail.com (D.C.d.C.); colaresluciana@gmail.com (L.P.C.L.); fernando.mellojr@hotmail.com (F.A.R.M.J.); alaydevieira@yahoo.com.br (A.V.W.); robertaborgesandrade@gmail.com (R.B.A.d.S.); acohencastro@gmail.com (A.C.-P.); fernandesmr@yahoo.com.br (M.R.F.); sidneysantos@ufpa.br (S.S.); khayatas@gmail.com (A.S.K.); assumpcaopp@gmail.com (P.P.d.A.); 2 Departamento de Pediatria, Ophir Loyola Hospital, Belém 66063-240, Brazil; 3 Human and Medical Genetics Laboratory, Instituto de Ciências Biológicas, Universidade Federal do Pará, Belém 66075-110, Brazil; xtatixsouza@gmail.com; 4 João de Barros Barreto University Hospital, Universidade Federal do Pará, Belém 66063-023, Brazil

**Keywords:** acute lymphoblastic leukemia, pediatrics, TPMT, polymorphism, pharmacogenetics, mortality, ancestry, admixed populations, Amazon

## Abstract

Acute lymphoblastic leukemia (ALL) is the leading cause of death from pediatric cancer worldwide. However, marked ethnic disparities are found in the treatment of childhood ALL with less effective results and higher mortality rates being obtained in populations with a high level of Native American ancestry. Genetic variations of the patient can affect resistance to ALL chemotherapy and potentially play an important role in this disparity. In the present study, we investigated the association of 16 genetic polymorphisms with the cell and metabolic pathways of the chemotherapeutic agents used in the treatment of ALL with the risk of death in treating childhood ALL in patients with a high contribution of Amerindian ancestry, coming from the Brazilian Amazon. The study included 121 patients with B-cell ALL treated with the BFM-2002 protocol. We are the first to identify the association between the TPMT gene rs1142345 polymorphism and the high risk of death in treating childhood ALL. Patients with the CC genotype had an approximately 25.5 times higher risk of dying during treatment of the disease than patients with other genotypes (*p* = 0.019). These results may help elucidate how the patient’s genetic characteristics contribute to the mortality disparity in populations with a high contribution of Native American ancestry. The rs1142345 variant of the TPMT gene could be used as a potential marker to early stratify patients at high risk of death in treating childhood ALL in the investigated population.

## 1. Introduction

Acute lymphoblastic leukemia (ALL) is the most common childhood cancer, and the principal cause of death from malignant diseases in childhood [1,2,3]. The chemotherapeutic drugs 6-mercaptopurine (6-MP) and methotrexate (MTX) are the principal compounds used for treating ALL, which helps increase survival rates by around 90% [4]. However, this treatment fails in 20% of pediatric patients, which results in high mortality rates [5,6,7].

One factor that may affect the success of ALL treatment is the patient’s ethnic background. Hispanic children with ALL treated using the same therapeutic regimens as those of European descent have higher mortality rates [8,9]. This indicates a differential role of genetic factors. A number of studies have concluded that the differential mortality rates in Hispanics with ALL are related to a genetic predisposition linked to the Native American ancestry of these populations [10,11,12,13,14]. In fact, many of the genetic variants associated with the toxicity and relapses in the treatment of ALL have been found more frequently in Native American populations or in populations with a high degree of miscegenation involving this ethnic group [15,16,17,18].

The miscegenated population of the Brazilian Amazon has the greatest contribution of Amer-Indian ancestry in the country with a mean of approximately 30% [19,20]. Our research group recently reported high rates of severe toxicity associated with specific variants of the genes responsible for the cellular and metabolic pathways of 6-MP and MTX [21], even though it remained unclear whether there is a link between these genetic variants and the risk of death associated with the treatment of the disease.

In this context, the identification of genetic markers associated with a greater risk of mortality during the treatment of childhood ALL will be important for evaluating groups with a major Amer-Indian component, such as the miscegenated population of the Amazon region. This will be potentially extremely valuable for the development of more personalized therapeutic strategies that reduce the risk of death in patients with specific alleles. The present study investigated the possible relationship between 16 genetic markers involved in the 6-MP and MTX pharmacological pathways, and the risk of death in pediatric patients with ALL in the Brazilian Amazon.

## 2. Materials and Methods 

### 2.1. Patients

This study included 121 patients with B-cell ALL diagnosed at two public hospitals between 2006 and 2017 (the Ophir Loyola Hospital and the Octavio Lobo Childhood Cancer Hospital) in the city of Belém, Pará (Brazil), which are reference institutions for treating childhood cancer in the Amazon region. The sampling plan adopted was based on selection by simple random sampling with a 95% confidence level. All the participants of the study had a similar socio-economic background and resided in the same geographic region. The patients were treated primarily with the protocol of the Berlin-Frankfurt-Münster international study group (ALL IC-BFM 2002) and were divided into standard, medium, and high-risk groups. The two drugs (MTX and 6-MP) were administered in the consolidation and maintenance phases. In the consolidation phase of the standard and medium risk patients, the M protocol was applied. This protocol consists of doses of 2000 mg/m^2^ of MTX and 25 mg/m^2^ of 6-MP. The HR protocol (5000 mg/m^2^ of MTX, without 6-MP) was applied in the case of high-risk patients. During the maintenance phase, the standard and medium risk patients received 20 mg/m^2^ of MTX and 50 mg/m^2^ of 6-MP, while the high-risk patients were treated using the St. Jude protocol, which consists of 40 mg/m^2^ of MTX and 75 mg/m^2^ of 6-MP (see Reference [21] for details of the treatment). The medical records of all the patients were analyzed, retrospectively, for clinical and historical data. Only deaths related to toxicity 3–4th degree arising during the consolidation and maintenance phases of the ALL treatment were included in the analysis. Patients do not have other pathogenic comorbidities. The protocol used for this study was approved by the Center for Ethics and Research in Humans (CER) of the Institute of Health Sciences (ICS) of the Federal University of Pará. The specific ethical code number is 119.649. The legal guardians of each patient included in the study signed a free and informed consent form.

### 2.2. Analysis of Genetic Ancestry and Cytogenetic Data

**Figure 1:**
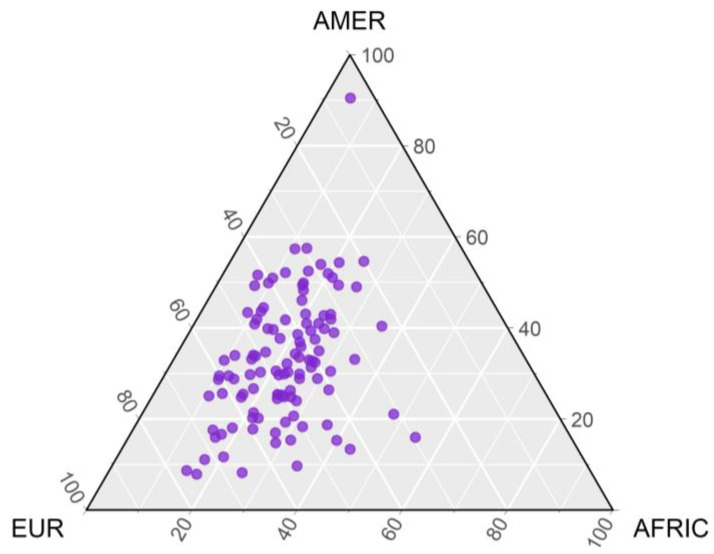
Representation of individuals’ inter-ethnic admixtures. Patients with ALL are represented by a dot in purple and their location on the graph corresponds to the mixture ratios. The mixture is estimated by comparison with parent populations of individuals represented in the triangle vertices: European (EUR), Amerindian (AMER), and African (AFRIC).

To demonstrate that the studied population is miscegenated with a high Amerindian contribution, the genetic ancestry of the patients included in the study was confirmed using the set of 61 Ancestry Informative Markers (AIMs) described in References [19,22]. All the individuals included in the present study had more than 30% Amer-Indian ancestry (Figure 1). The proportions of European, African, and Native American ancestries were estimated using STRUCTURE v2.3.3 by assuming the existence of three parental populations (European, African, and Amerindian). Parental populations included 270 European individuals (mainly Portuguese individuals), 211 Sub-Saharan Africans (individuals from Angola, Mozambique, Zaire, Cameroon, and the Ivory Coast), and 222 Native American individuals (from indigenous tribes of the Brazilian Amazon region). Details about these populations can be found in Reference [19].

Cytogenetic data defining the gene fusions of five ALL subtypes—BCR-ABL, ETV6-RUNX1, MLL-AF4, SIL-TAL, and TCF3-PBX1—were obtained for 99 of the B-cell ALL patients included in the study, with the analyses being conducted by RT-PCR, as described by Reference [23].

### 2.3. Selection of the SNPs, Extraction and Quantification of the DNA, and Genotyping

Sixteen polymorphic genes were selected for the present study. These genes are involved primarily in the metabolic pathways of the MTX and 6-MP drugs used in the ALL IC-BFM-2002 protocol for the treatment of B-cell ALL (Table 1). The genes were selected based on the PharmGKB, NCBI, and Ensembl databases, and the data available in previous studies. Peripheral blood was obtained from the ALL patients during routine sample collection for blood counts. The genetic material was extracted from these blood samples using the Roche Applied Science DNA extraction kit (Roche, Penzberg, Germany), which followed the manufacturer’s instructions, and was quantified using a NanoDrop 1000 spectrophotometer (NanoDrop Technologies, Wilmington, DE, USA). The polymorphisms were genotyped using the QuantStudio™12K Flex Real-Time PCR system by TaqMan Open Array Genotyping (Applied Biosystems, Life Technologies, Carlsbad, CA, USA) by following the protocol that Applied Biosystems published. 

### 2.4. Statistical Analysis

The statistical analyses were run in SPSS v.25.0. The first step was to determine whether each polymorphism was in the Hardy-Weinberg Equilibrium (HWE) using Chi-square. Five variants (rs717620, rs1801394, rs4149056, rs1800462, and rs56161402) were not in HWE, and were excluded from the analysis while leaving 11 polymorphisms for the analysis of association. The occurrence of death was related to the characteristics of the patient at initial diagnosis, that is, their sex, age at diagnosis (<10 or ≥10 years), initial leukocyte count (<50 × 109/L or ≥50 × 109/L), cytogenetic data, risk group (standard, medium, and high), and genetic ancestry (European, Amer-Indian, and African). The variation in categorical variables was tested using Chi-square, while the continuous variables were analyzed using Student’s t-test and Mann-Whitney’s U-test. The influence of the genetic variants on the risk of death during the treatment of the disease was evaluated using a multivariate logistic regression, which included different groups of risk, to control for confounding effects. All statistical tests were two-tailed and a significance level of *p* ≤ 0.05.

## 3. Results

The clinical data on the 121 B-cell patients included in the present study are provided in Table 2. The mean age of the patients was 5.29 ± 3.32 years, and the majority were male (59.5%). The participants of the study had a high proportion of Amer-Indian ancestry with a mean of 33% (Figure 1).

In the case of the cytogenetic parameters, 46.5% of the patients lacked any of the target translocations. The most frequent fusion was TCF3-PBX1, which was followed by BCR-ABL and ETV6-RUNX1. Thirty-five (28.9%) of the ALL patients died during treatment, and the analysis of their clinical variables (Table 3) revealed significant differences in the standard (*p* = 0.001) and the high risk (*p* < 0.001) group. Given this, these variables were controlled for in the multivariate logistic regression.

The influence of the 11 polymorphisms included in this analysis on mortality in treating childhood ALL is shown in Table 4. In particular, the rs1142345 polymorphism of the TPMT gene was associated significantly with the risk of death in treating the disease. Patients with genotype (CC) of this variant had an approximately 25.5 times greater risk of death during ALL treatment than those with other genotypes (*p* = 0.019, OR = 25.446, 95% CI = 1.698–381.402). 

We tested whether the two variants of the *TPMT* gene included in the study (rs1800460, defining the *3C allele and rs1142345, defining the *3B allele) were allelic to each other, and constituted the *3A allele (rs1800460 and rs1142345). We found one patient (0.8%) with the *3A/*3B genotype and 10 patients (8.3%) with the *3A/*1 genotype. These haplotypes are related to the low and intermediate 6-MP metabolization phenotypes, respectively. They were not statistically significant in the analysis.

The analyses found no significant pattern in any of the other polymorphisms analyzed in the study.

**Figure 2:**
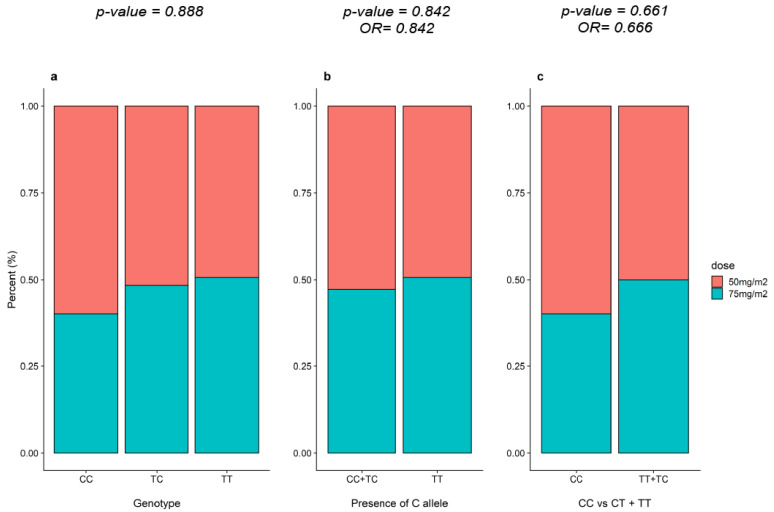
Distribution of TPMT_rs1142345 variants according to the 6-MP dose used in the maintenance phase of the ALL treatment (**a**) between carriers and non-carriers of the C allele (**b**) and among patients with genotype CC vc CT + CC (**c**).

Additional analysis was carried out to elucidate whether the dose of 6-MP administered in the maintenance of ALL therapy differed in carriers of the C allele and non-carriers or subjects with and without the TPMT *3C variant. According to the results presented in Figure 2, the doses of 6-MP did not differ significantly among patients with the presence of the C allele (Figure 2b) or among patients with the TPMT *3C genotype and those without (Figure 2c).

## 4. Discussion

Although survival rates in ALL patients have improved considerably in developed countries in recent decades, survival rates are still low in underdeveloped and developing nations, which range from 30% to 70% [5]. A number of prognostic factors can affect survival in ALL treatment, including the age of the patient, their leukocyte count, immunophenotyping, and cytogenetics. These factors are incorporated into the clinical protocols to allocate the patients to standard, medium, and high-risk groups [24]. Even with treatment regimens adapted to these clinical risk factors, ethnic disparities persist in the treatment of ALL with Hispanic children continuing to present a worse therapeutic outcome and higher mortality rates than patients of other ethnicities [8,9,10,11,12,13,14].

In the present study, we recorded a mortality rate of almost 30% in the ALL patients with the highest rate being observed in the high-risk group. Interestingly, common chromosomal alterations that are known to be highly prognostic in ALL, such as BCR-ABL, ETV6-RUNX1, MLL-AF4, SIL-TAL, and TCF3-PBX1, were not related to the risk of death in the patients investigated in the present study. These chromosomal changes are not associated with the patient’s ethnicity in general [9,12].

Previous studies have shown that pediatric patients of Latin origin diagnosed with ALL tend to have more high-risk genetic characteristics [12,25,26,27]. However, clinical factors and the biology of the tumor alone are insufficient to account for the disparity in the outcomes recorded in Hispanic children with the worst results being related to the chemotherapeutic response [27,28]. Clearly, the genetics of the patient may affect their resistance to chemotherapy or the pharmacokinetics of agents such as MTX and 6-MP [29,30,31,32].

Given this, we investigated the potential role of genetic variants linked to the metabolic pathways of MTX and 6-MP in the risk of death during the ALL treatment of pediatric patients with a high level of Amer-Indian ancestry. Our findings show that patients carrying the CC genotype of the rs1142345 variant of the *TPMT* gene had an approximately 25.5 higher risk of dying during ALL treatment than those with other alleles. 

The *TPMT* gene is the principal regulator of the balance between active and inactive 6-MP metabolites, and the TPMT*3C (rs1142345) polymorphism alters the expression of the gene at the mRNA level, which makes the protein more susceptible to degradation and leads to a reduction in the inactivation of the 6-MP drug [33]. This makes the carries of the CC genotype of TPMT*3C variant susceptible to toxicity from standard doses of 6-MP. Studies of a number of different populations have found an association between the TPMT*3C variant and toxicity resulting from 6-MP treatment in ALL patients [34,35,36,37,38]. As far as we are aware, however, this is the first study to associate this polymorphism with an increased risk of death in treating childhood ALL. 

Our research group recently identified a high frequency of the CC genotype of the TPMT*3C variant in traditional indigenous populations of the Amazon region. This frequency was significantly different from those recorded in other populations worldwide [17], which clearly indicates that this polymorphism is more frequent in Amer-Indian populations and in populations admixed with these groups, as the population investigated in the present study. These findings reinforce the conclusion that pharmacogenetic data obtained from homogeneous ethnic groups cannot be applied to highly admixed populations.

In our study, we found no statistically significant differences for other variants in the *TPMT* gene (*3B and *3A allele) in the risk of death for treating childhood ALL. The literature reports that the rs1142345 variant of the *TPMT* gene appears to be of greater clinical importance compared to the other variants of the gene. In a study from Liu et al. [35] involving ethnically diverse cohorts of pediatric patients with ALL, through agnostic GWAS approaches to assess the contribution of genetic variation of TPMT and other genes to the activity and effects of the TPMT enzyme on tolerance to thiopurine, the *TPMT* gene alone reached significance in the entire genome with the rs1142345 variant in the top hit. In the same study, the C allele was associated with exposure to mercaptopurine in children with leukemia when compared to the T allele. In addition, in our study, patients with the TC genotype of the rs1142345 variant of the *TPMT* gene were not associated with the risk of death in treating disease. Despite the literature reporting that patients with the TC genotype may show increased toxicity when treated with mercaptopurine and require a reduced dose of 6-MP compared to patients with TT genotypes [39,40], the clinical toxicological manifestations in relation to heterozygous patients are not unanimous when investigating different populations in the world. In a study involving Near Eastern populations [41], the heterozygous genotype was not associated with the dose of mercaptopurine in children with ALL. In another study [42] involving mixed populations, only the CC genotype was associated with a decreased dose of mercaptopurine in children with ALL when compared to the TC and TT genotypes.

Since this is the first result, larger studies should be carried out to elucidate the role of the TPMT *3C variant in the risk of death in treating ALL in other populations miscegenated with Amer-Indian groups. We believe that our study can assist future investigations, and work as an alert for studies in this field.

## 5. Conclusions

The present study revealed the first finding of a significant association between the rs1142345 variant of the *TPMT* gene and an increased risk of death in treating pediatric ALL. The results of the present study provide important insights for the identification of groups of individuals that are more susceptible to death when treating childhood ALL, in addition to contributing toward understanding the influence of genetic characteristics on the therapeutic outcome of ALL treatment in populations with a high contribution of Amer-Indian ancestry.

## Figures and Tables

**Table 1:** Characterization of the polymorphisms analyzed in the present study.

Gene	SNP	Allele	Function	Amino Acid Substitution	Chromosome	Location	HWE ^a^
*ABCC1*	rs28364006	A > G	Missense	Thr1337Ala	16	16134392	Accepted
*ABCC2*	rs717620	C > T	5′ UTR	-	10	99782821	No HWE
*ABCC3*	rs9895420	T > A	5′ Flanking	-	17	50634677	Accepted
*ATIC*	rs2372536	C > G	Missense	Thr116Ser	2	215325297	Accepted
*ATIC*	rs4673993	T > C	Splicing region	-	2	215347616	Accepted
*ITPA*	rs1127354	C > A	Missense	Pro15Thr	20	3213196	Accepted
*MTHFD1*	rs2236225	G > A	Missense	Arg653Gln	14	64442127	Accepted
*MTHFR*	rs1801133	G > A	Missense	Ala222Val	1	11796321	Accepted
*MTRR*	rs1801394	A > G	Missense	Ile22Met	5	7870860	No HWE
*SLCO1B1*	rs2306283	A > G	Missense	Asn130Asp	12	21176804	Accepted
*SLCO1B1*	rs4149015	G > A	5′ Flanking	-	12	21130388	Accepted
*SLCO1B1*	rs4149056	T > C	Missense	Val174Ala	12	21178615	No HWE
*TPMT*	rs1800460	C > T	Missense	Ala154Thr	6	18138997	Accepted
*TPMT*	rs1800462	C > G	Missense	Ala80Pro	6	18143724	No HWE
*TPMT*	rs1142345	T > C	Missense	Tyr240Cys	6	18130687	Accepted
*TPMT*	rs56161402	C > T	Missense	Arg215His	6	18130762	No HWE

Abbreviations: 3UTR: 3′ UTR regulation. 5UTR: 5′ UTR regulation. ^a^ *p*-value adjusted by a Bonferroni correction.

**Table 2:** Clinical characteristics of patients analyzed in the present study.

Characteristics	N^o^ of Subjects and Frequency (%)
Gender	
Male	72 (59.5)
Female	49 (40.5)
Mean age at diagnosis (years, SD±)	5.29 ± 3.32
Age at diagnosis (years)	
<10	103 (85.1)
≥10	18 (14.9)
Leukocyte count at diagnosis/μL	
<50,000	94 (77.7)
≥50,000	27 (22.3)
Chromosomal translocations	99
Absent	46 (46.5)
*BCR-ABL*	16 (16.2)
*ETV6-RUNX1*	12 (12.1)
*MLL-AF4*	1 (1.0)
*TCF3-PBX1*	23 (23.2)
*SIL-TAL*	1 (1.0)
Risk group	
Standard	50 (41.3)
Medium	11 (9.1)
High	60 (49.6)
Death frequency	
Yes	35 (28.9)
No	86 (71.1)
Genetic Ancestry (SD±)	
European	0.45 ± 0.10
Amer-Indian	0.33 ± 0.11
African	0.22 ± 0.07

Abbreviations: SD, Standard Deviation.

**Table 3:** Clinical characteristics of the patients by the death rate in the treatment of childhood ALL.

Characteristics	N^o^ of Subjects and Frequency (%)		*p*
	Death	Non-Death	
Gender			0.088
Male	25 (71.4)	47 (54.7)	
Female	10 (28.6)	39 (45.3)	
Age at diagnosis (years, SD±)	5.20 ± 3.52	5.33 ± 3.26	0.907
<10	30 (85.7)	73 (84.9)	
≥10	5 (14.3)	13 (15.1)	
Leukocyte count at diagnosis/μL			0.124
<50,000	24 (68.6)	70 (81.4)	
≥50,000	11 (31.4)	16 (18.6)	
Chromosomal translocations (n = 99)			0.530
*Absent*	11 (44)	35 (47.3)	
*BCR-ABL*	4 (16)	12 (16.2)	
*ETV6-RUNX1*	2 (8)	10 (13.5)	
*MLL-AF4*	0	1 (1.4)	
*TCF3-PBX1*	7 (28)	16 (21.6)	
*SIL-TAL*	1 (4)	0 (1.4)	
Risk group			
Standard	6 (17.1)	44 (51.1)	0.001
Medium	2 (5.8)	9 (10.5)	0.410
High	27 (77.1)	33 (38.4)	<0.001
Genetic Ancestry (SD±)			
European	0.45 ± 0.11	0.46 ± 0.12	0.606
Amer-Indian	0.32 ± 0.11	0.34 ± 0.14	0.546
African	0.23 ± 0.10	0.20 ± 0.07	0.249

**Table 4:** Association analysis of selected generic variants and the incidence of death in treating ALL in childhood.

Gene_SNP	Genotype	Death n (%)	Non-Death n (%)	*p* ^a^	OR (95% CI) ^a^
*ABCC1_*rs28364006				0.339	GG vs. others: 0.685 (0.603–0.777)
	AA	32 (91.4)	71 (91)		
	AG	3 (8.6)	5 (6.4)		
	GG	0	2 (2.6)		
*ABCC3_*rs9895420				0.148	AA vs. others: 0.205 (0.024–1.751)
	TT	31 (88.6)	67 (77.9)		
	TA	4 (11.4)	16 (18.6)		
	AA	0	3 (3.5)		
*ATIC_*rs2372536				0.248	GG vs. others: 0.486 (0.143–1.653)
	CC	19 (54.3)	37 (44)		
	CG	12 (34.3)	28 (33.3)		
	GG	4 (11.4)	19 (22.6)		
*ATIC_*rs4673993				0.767	CC vs. others: 1.186 (0.384–3.667)
	TT	18 (51.4)	43 (50)		
	TC	11 (31.4)	29 (33.7)		
	CC	6 (17.1)	14 (16.3)		
*ITPA_*rs1127354				0.524	AA vs. others: 0.711 (0.632–0.799)
	CC	32 (97)	76 (96.3)		
	CA	1 (3)	2 (2.4)		
	AA	0	1 (1.2)		
*MTHFD1_*rs2236225				0.447	AA vs. others: 1.504 (0.526–4.298)
	GG	15 (42.9)	32 (37.2)		
	GA	12 (34.3)	35 (40.7)		
	AA	8 (22.9)	19 (22.1)		
*MTHFR_*rs1801133				0.425	AA vs. others: 0.640 (0.214–1.913)
	GG	20 (57.1)	35 (40.7)		
	GA	9 (25.7)	33 (38.4)		
	AA	6 (17.1)	18 (20.9)		
*SLCO1B1_*rs2306283				0.208	GG vs. others: 1.849 (0.710–4.813)
	AA	11 (31.4)	24 (28.2)		
	AG	13 (37.1)	42 (49.4)		
	GG	11 (31.4)	19 (22.4)		
*SLCO1B1_*rs4149015				0.218	AA vs. others: 0.255 (0.029–2.242)
	GG	26 (74.3)	59 (70.2)		
	GA	8 (22.9)	16 (19)		
	AA	1 (2,9)	9 (10.7)		
*TPMT_*rs1800460				0.974	TT vs. others: 1.042 (0.088–12.339)
	CC	24 (88.9)	52 (78.8)		
	CT	2 (7.4)	11 (16.7)		
	TT	1 (3.7)	3 (4.5)		
*TPMT_* rs1142345				0.019	CC vs. others: 25.446 (1.698–381.402)
	TT	22 (62.9)	61 (72.6)		
	TC	9 (25.7)	22 (26.2)		
	CC	4 (11.4)	1 (1.2)		

^a^ Logistic regression adjusted for the risk group.
